# Supplementary material for: The actomyosin system is essential for the integrity of the endosomal system in bloodstream form Trypanosoma brucei
Source: eLife. 2024 Nov 21;13:RP96953. doi: 10.7554/eLife.96953 (PMC11581428; doi:10.7554/eLife.96953)
Supplement: Figure 1—source data 1. [file elife-96953-fig1-data1.zip › For zipping/Figure1-Source data1.pdf]

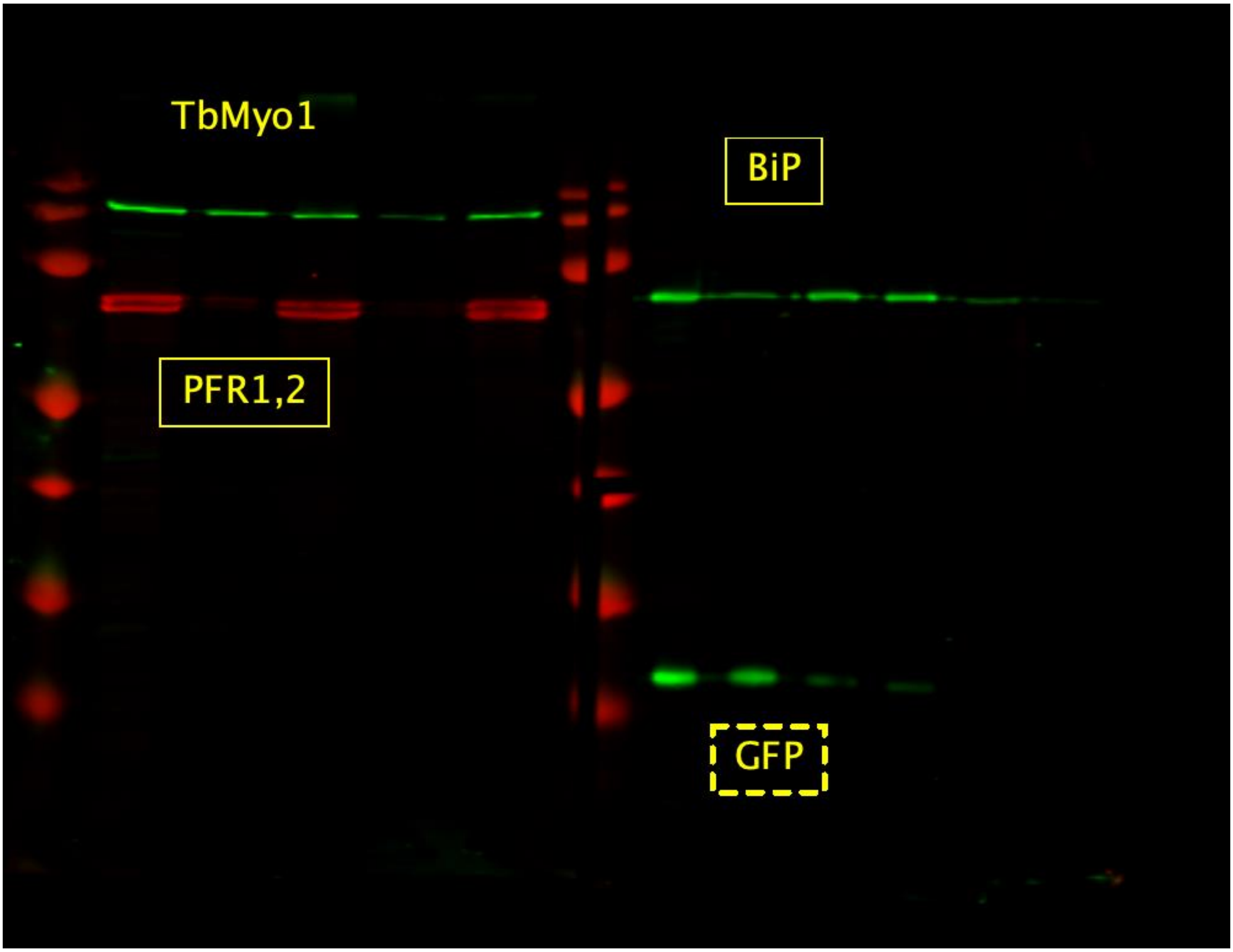

Panel B
Single membrane cut into three parts for immunoblotting.
All 3 Figure 3B panels taken from this immunoblot.
