# Supplementary figures and images for: The actomyosin system is essential for the integrity of the endosomal system in bloodstream form Trypanosoma brucei
Source: eLife. 2024 Nov 21;13:RP96953. doi: 10.7554/eLife.96953 (PMC11581428; doi:10.7554/eLife.96953)

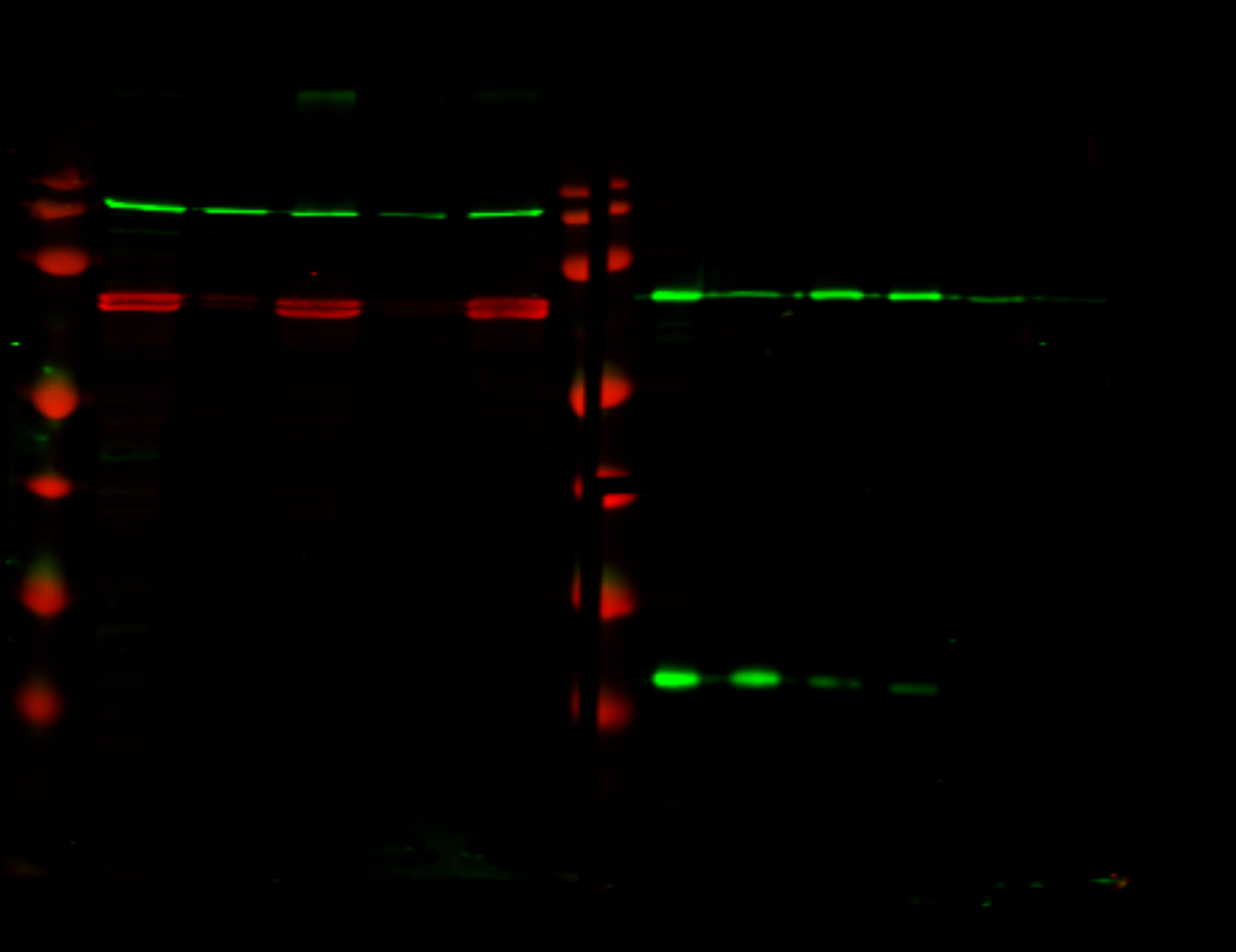

Supplement: Figure 1—source data 2. [file elife-96953-fig1-data2.zip › Figure1-source data2/Panel B__fractionation.tif]

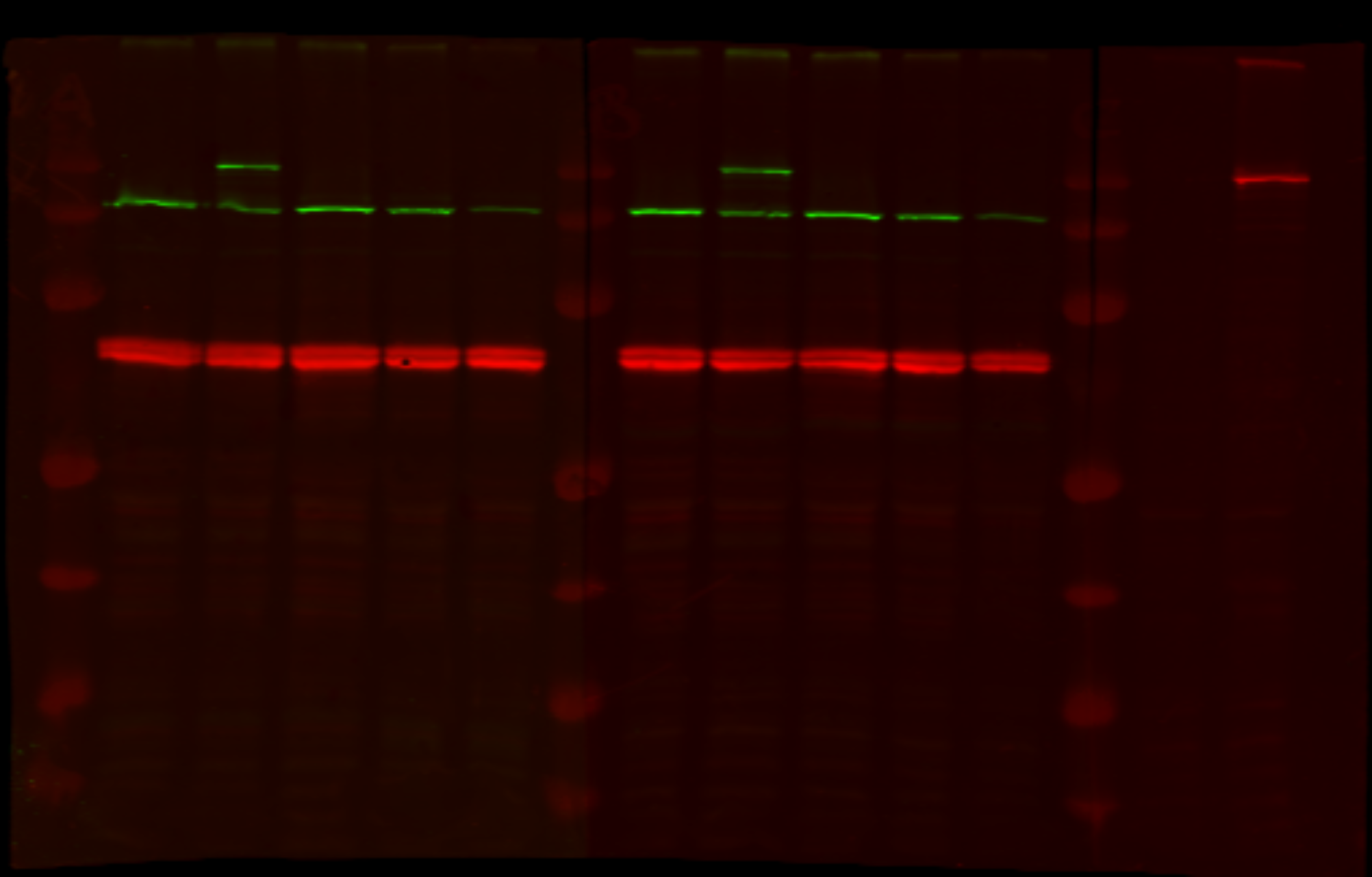

Supplement: Figure 1—figure supplement 1—source data 2. [file elife-96953-fig1-figsupp1-data2.zip › Figure1-Figure supplement 1-Source data2/panelD_TbMyo1_final blot1_20220214.tif]

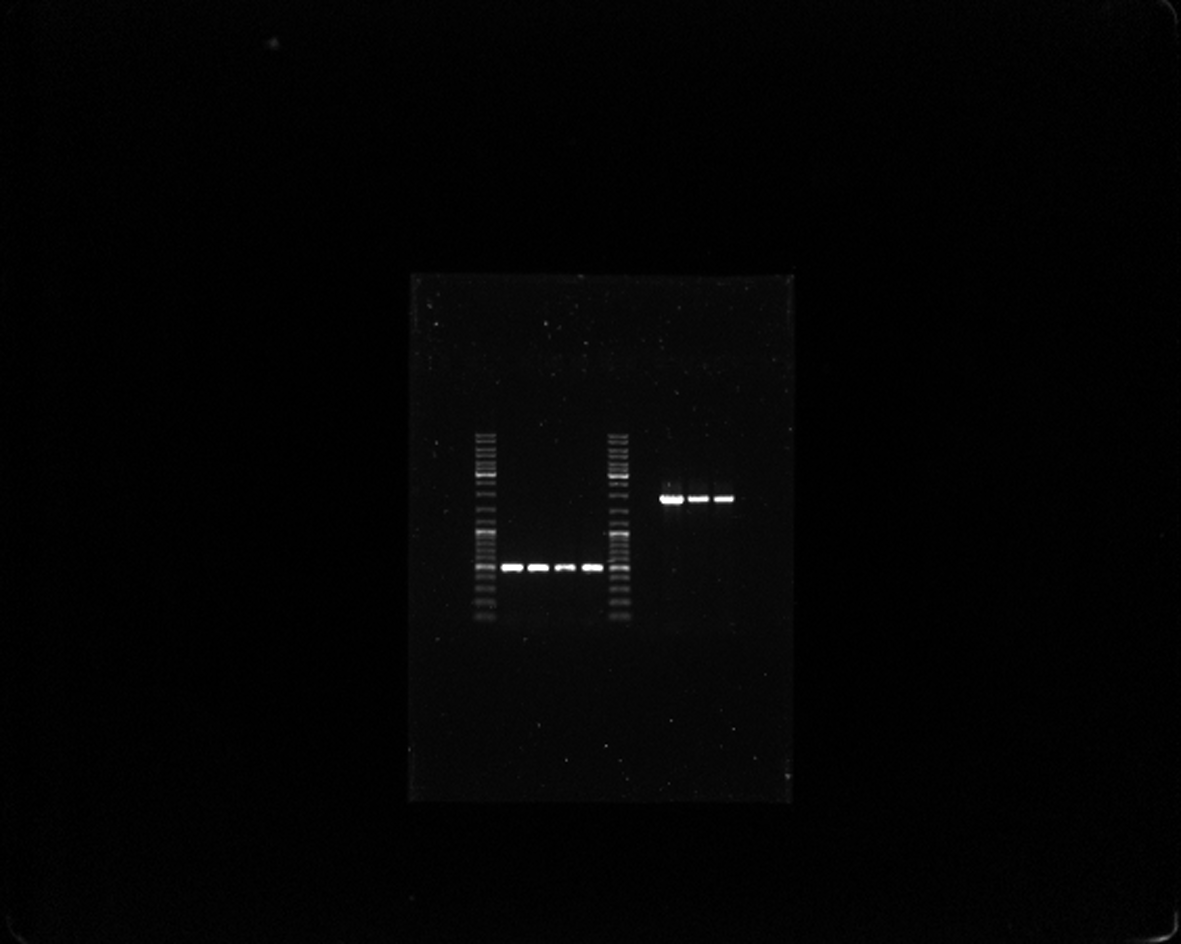

Supplement: Figure 1—figure supplement 1—source data 2. [file elife-96953-fig1-figsupp1-data2.zip › Figure1-Figure supplement 1-Source data2/panel B_original scan_gDNA_PCR_QuickLoad3_4ul.tif]

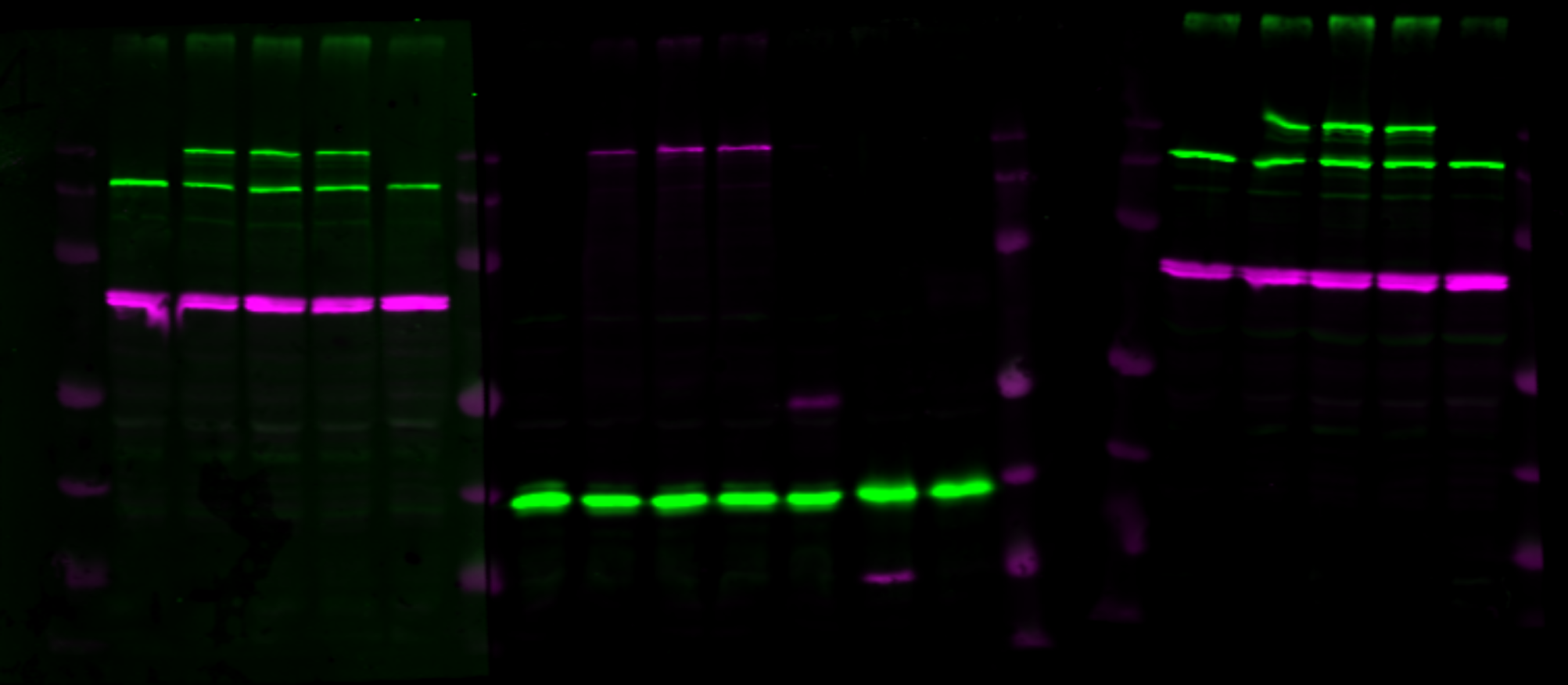

Supplement: Figure 1—figure supplement 1—source data 2. [file elife-96953-fig1-figsupp1-data2.zip › Figure1-Figure supplement 1-Source data2/panel C_complete scan_Blot-TbMyo1-Test.tif]

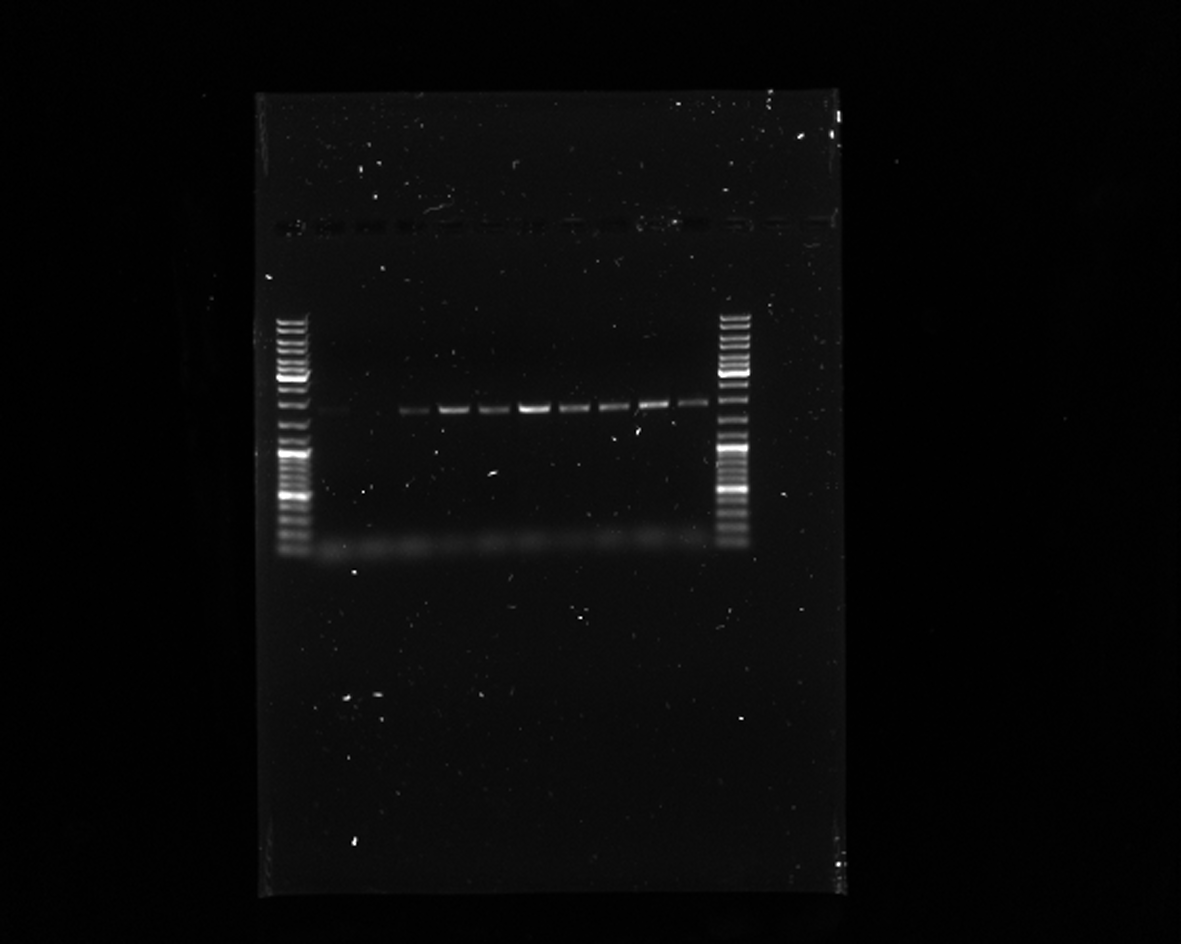

Supplement: Figure 1—figure supplement 2—source data 2. [file elife-96953-fig1-figsupp2-data2.zip › Figure1-Figure supplement 2-Source data2/Panel B_original scan_2022_02_22_125600.tif]

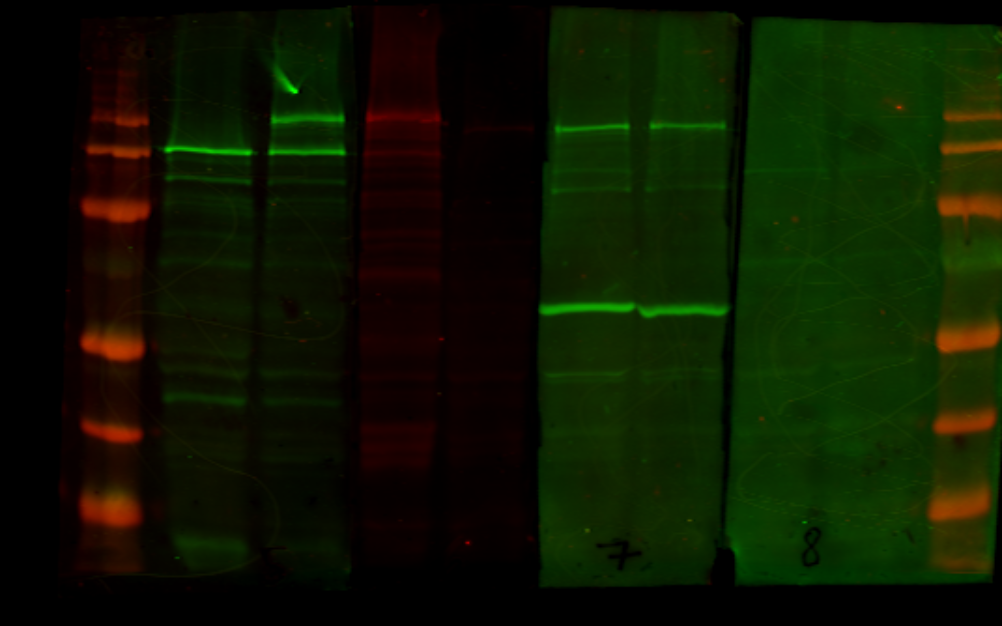

Supplement: Figure 1—figure supplement 2—source data 2. [file elife-96953-fig1-figsupp2-data2.zip › Figure1-Figure supplement 2-Source data2/Panel C_original scan_2022_04_22_mNG-Myo1 vs mNG-Myo21.tif]

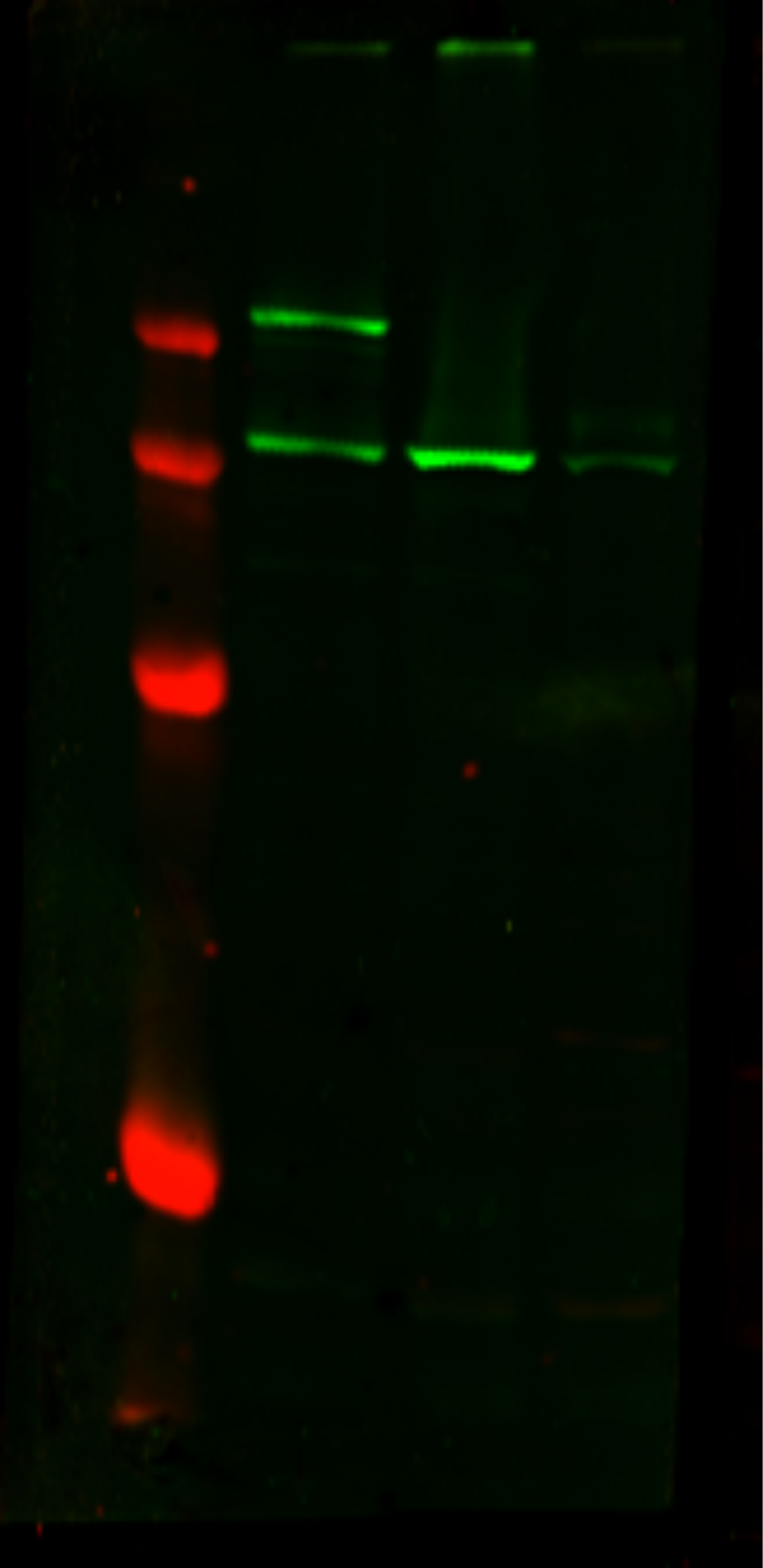

Supplement: Figure 1—figure supplement 2—source data 2. [file elife-96953-fig1-figsupp2-data2.zip › Figure1-Figure supplement 2-Source data2/Panel E_mNG-Myo21 diff blot_15-well_Myo1.tif]

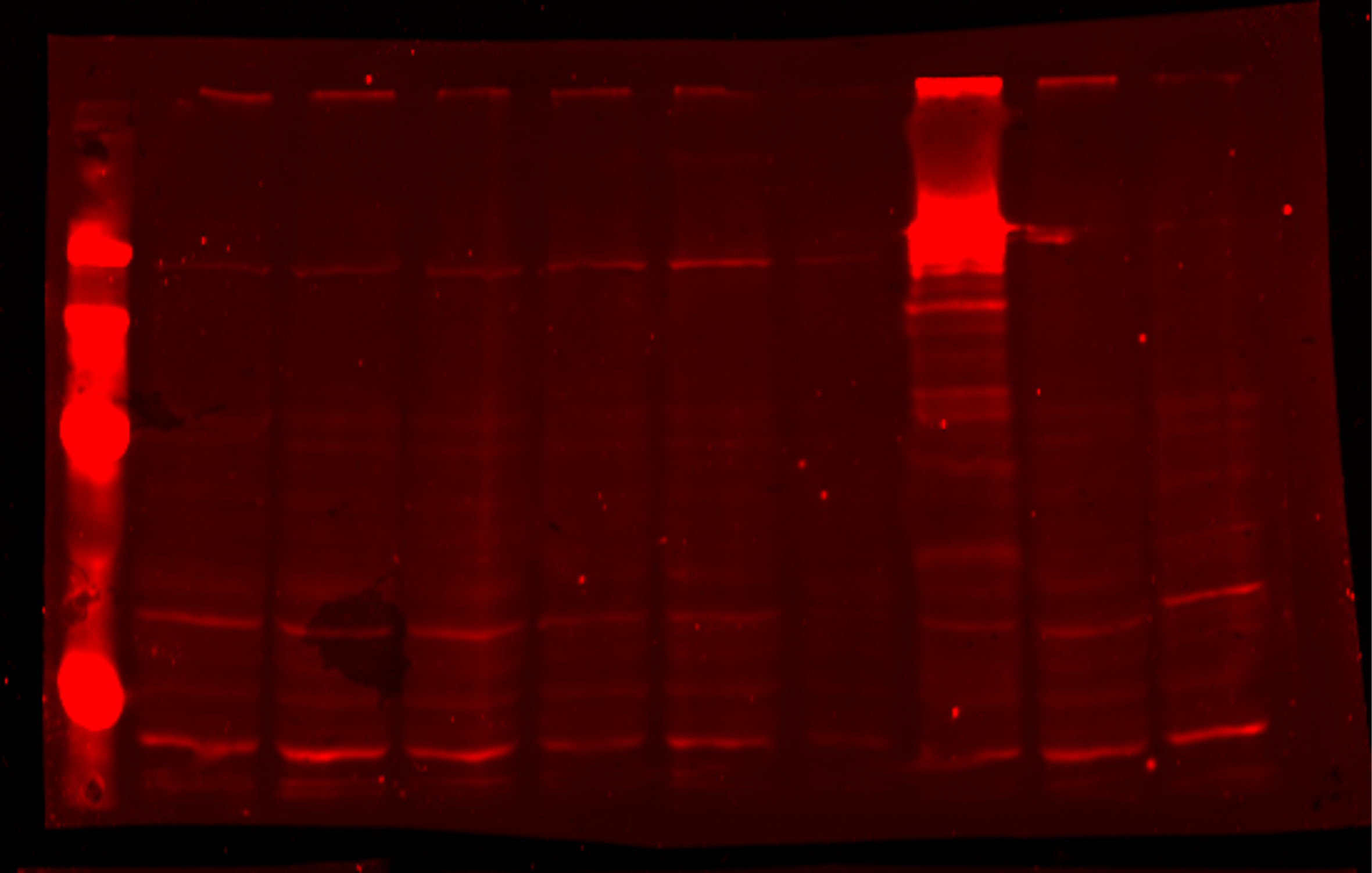

Supplement: Figure 1—figure supplement 2—source data 2. [file elife-96953-fig1-figsupp2-data2.zip › Figure1-Figure supplement 2-Source data2/Panel D_mNG-Myo21 diff blot_10-well_long expo.tif]

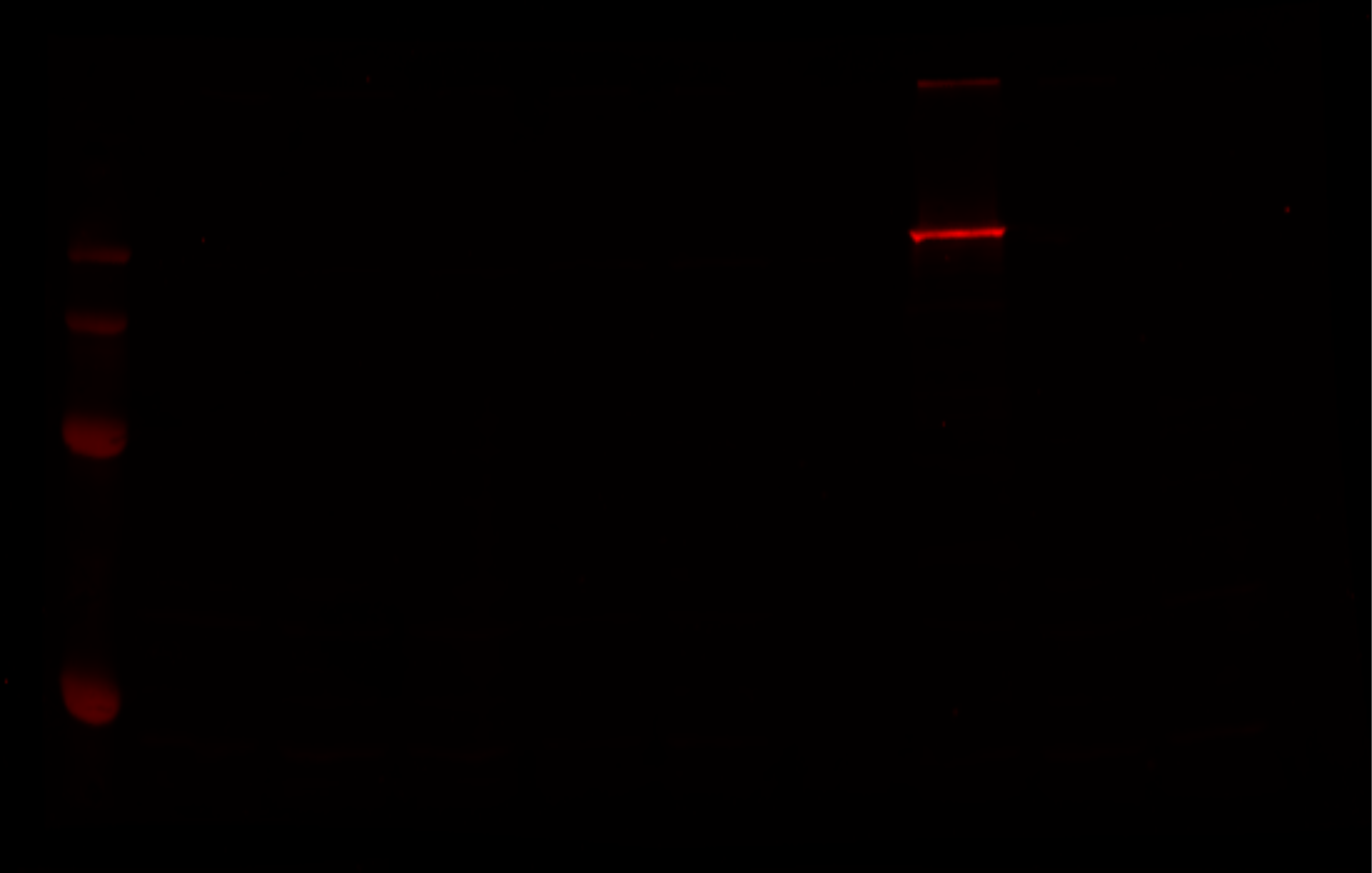

Supplement: Figure 1—figure supplement 2—source data 2. [file elife-96953-fig1-figsupp2-data2.zip › Figure1-Figure supplement 2-Source data2/Panel D_mNG-Myo21 diff blot_10-well_short expo.tif]

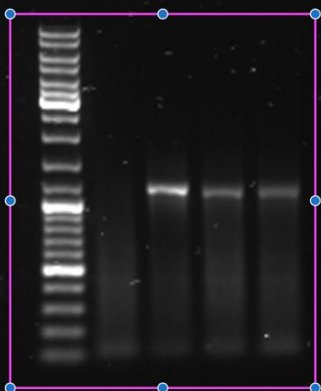

Panel A  
Display area outlined  
in magenta

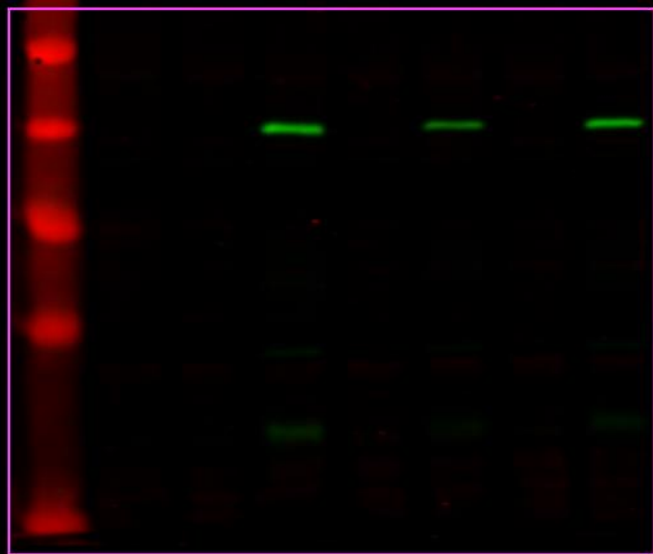

Panel B  
Display area outlined  
in magenta

Supplement: Figure 6—figure supplement 1—source data 1. [file elife-96953-fig6-figsupp1-data1.zip › For zipping/Figure6-Figure supplement1-Source data1.pdf]

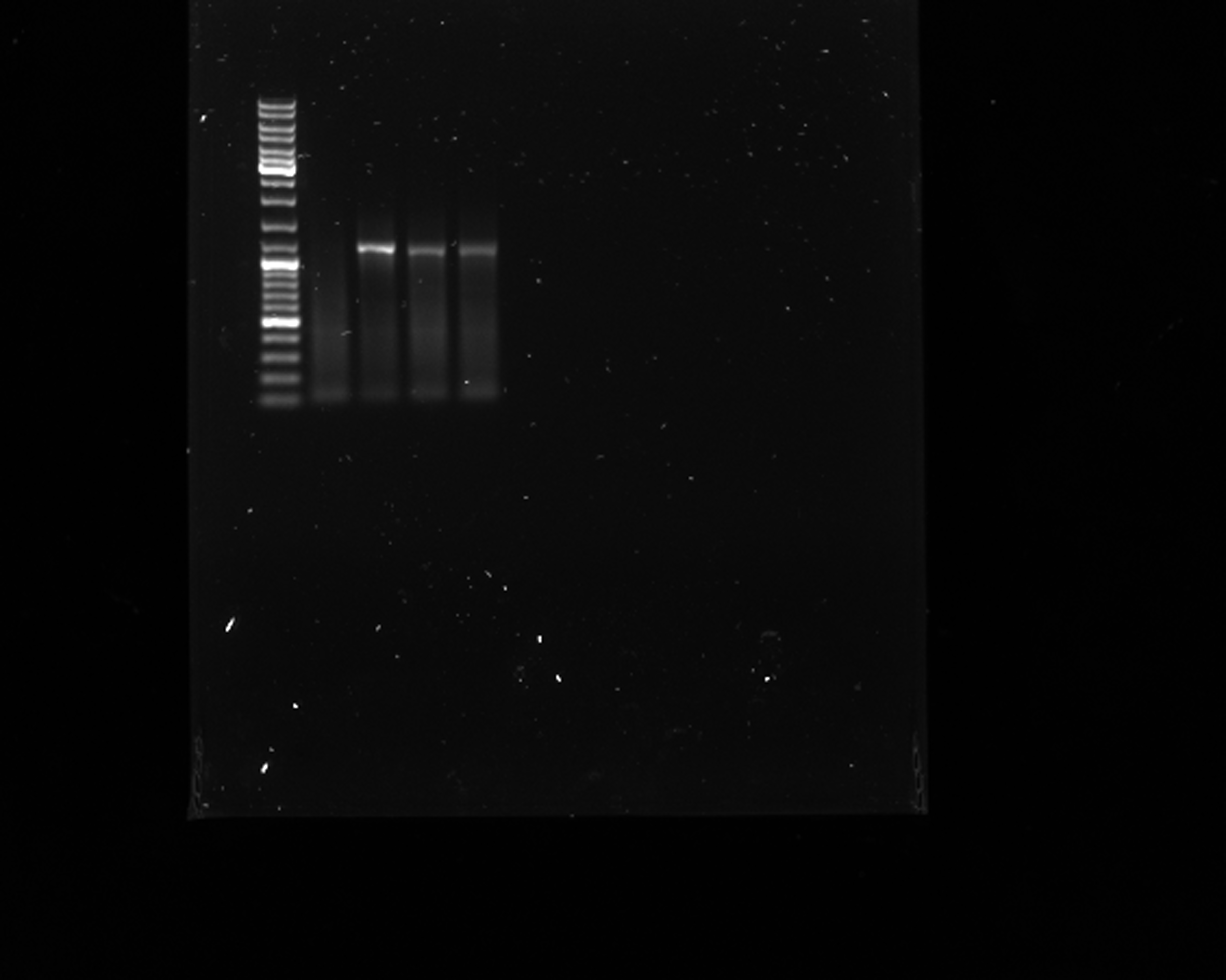

Supplement: Figure 6—figure supplement 1—source data 2. [file elife-96953-fig6-figsupp1-data2.zip › Figure6-Figure supplement1-Source data2/Panel A_2022_06_10_151145.tif]

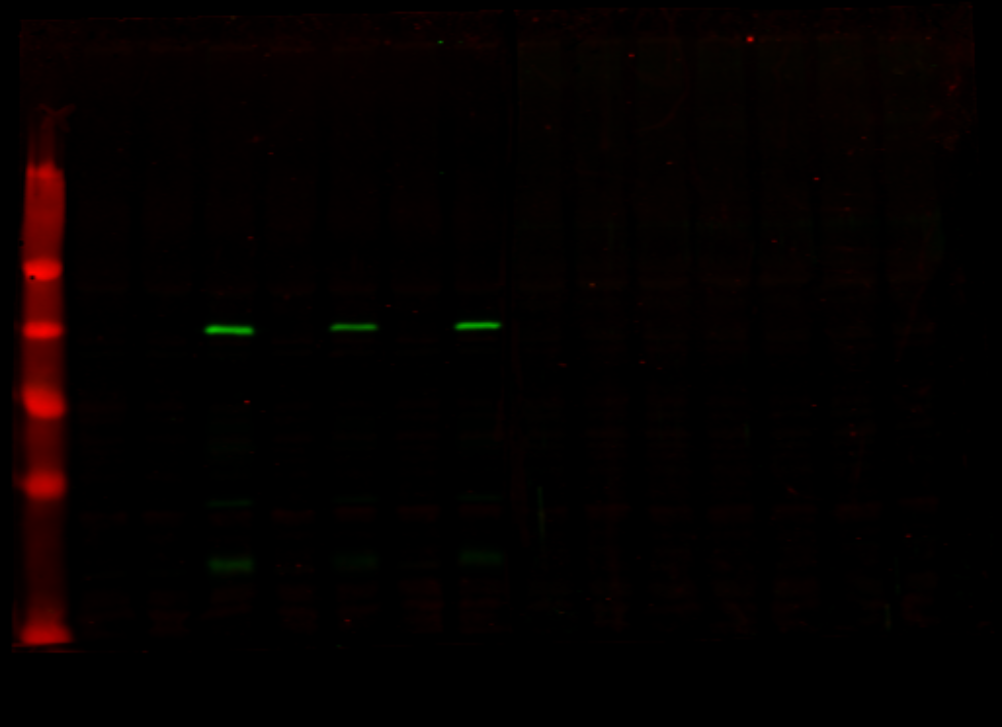

Supplement: Figure 6—figure supplement 1—source data 2. [file elife-96953-fig6-figsupp1-data2.zip › Figure6-Figure supplement1-Source data2/Panel B_2022_6.9_anti-RFP_1.tif]

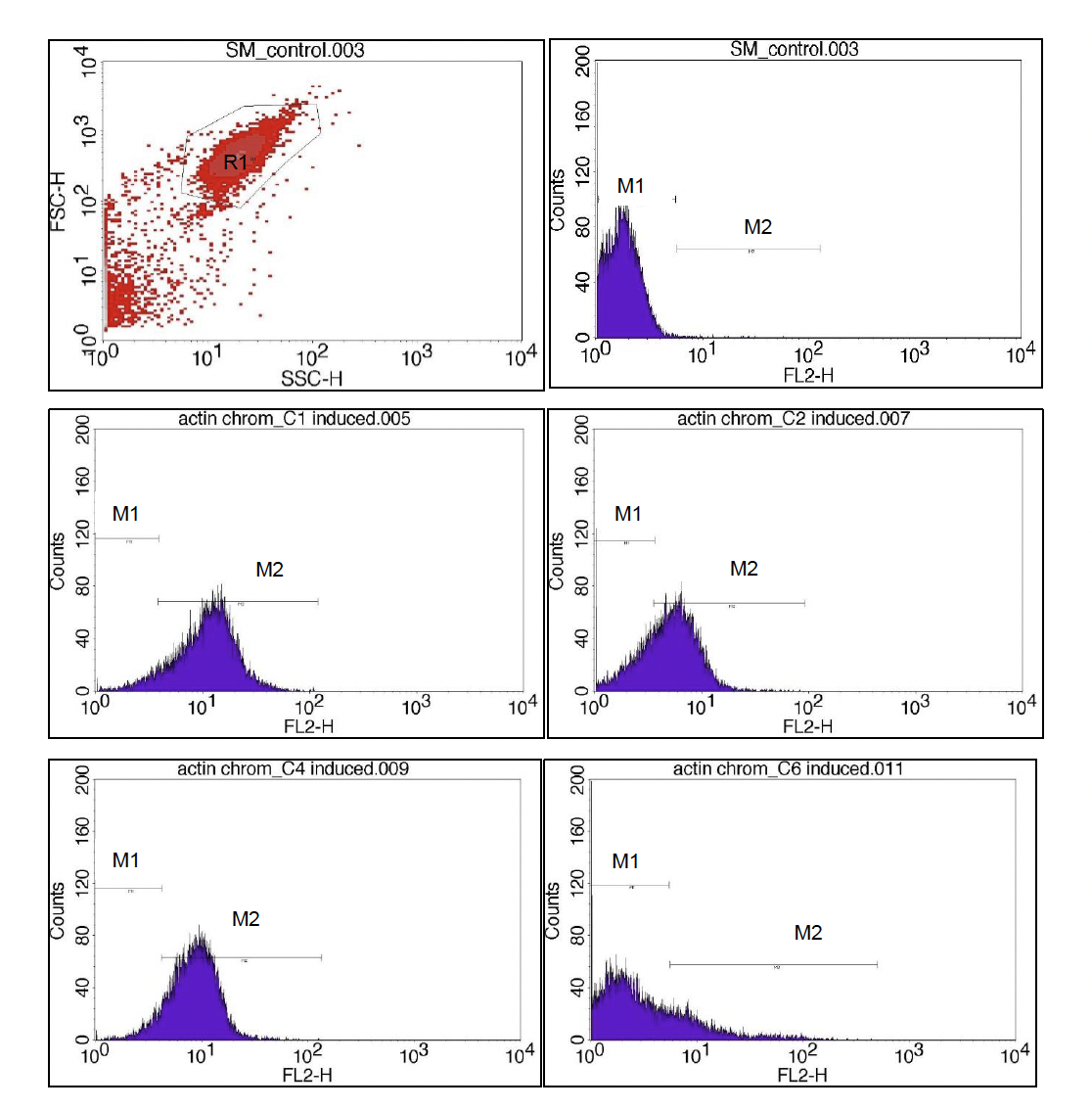

Supplement: Figure 6—figure supplement 1—source data 2. [file elife-96953-fig6-figsupp1-data2.zip › Figure6-Figure supplement1-Source data2/Panel C_Flow cytometry/Screenshot 2023-03-10 at 10.24.36.png]

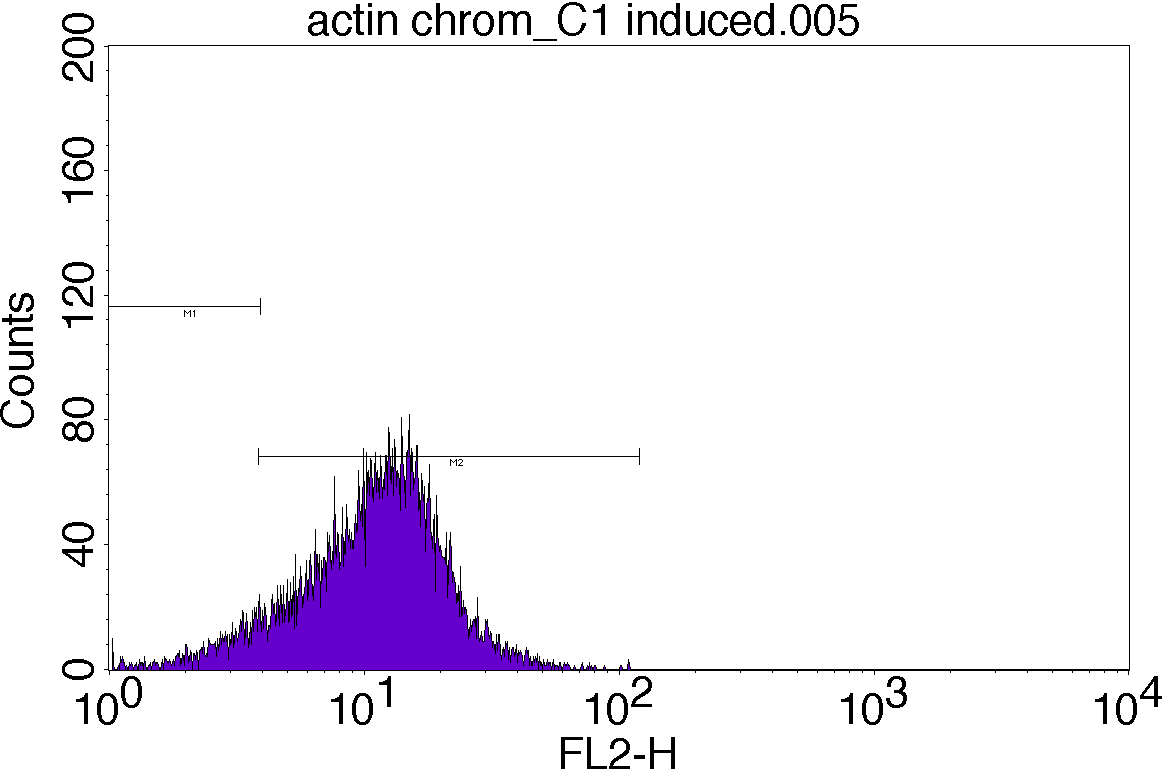

Supplement: Figure 6—figure supplement 1—source data 2. [file elife-96953-fig6-figsupp1-data2.zip › Figure6-Figure supplement1-Source data2/Panel C_Flow cytometry/actin chrom_C1 induced05cou.PNG]

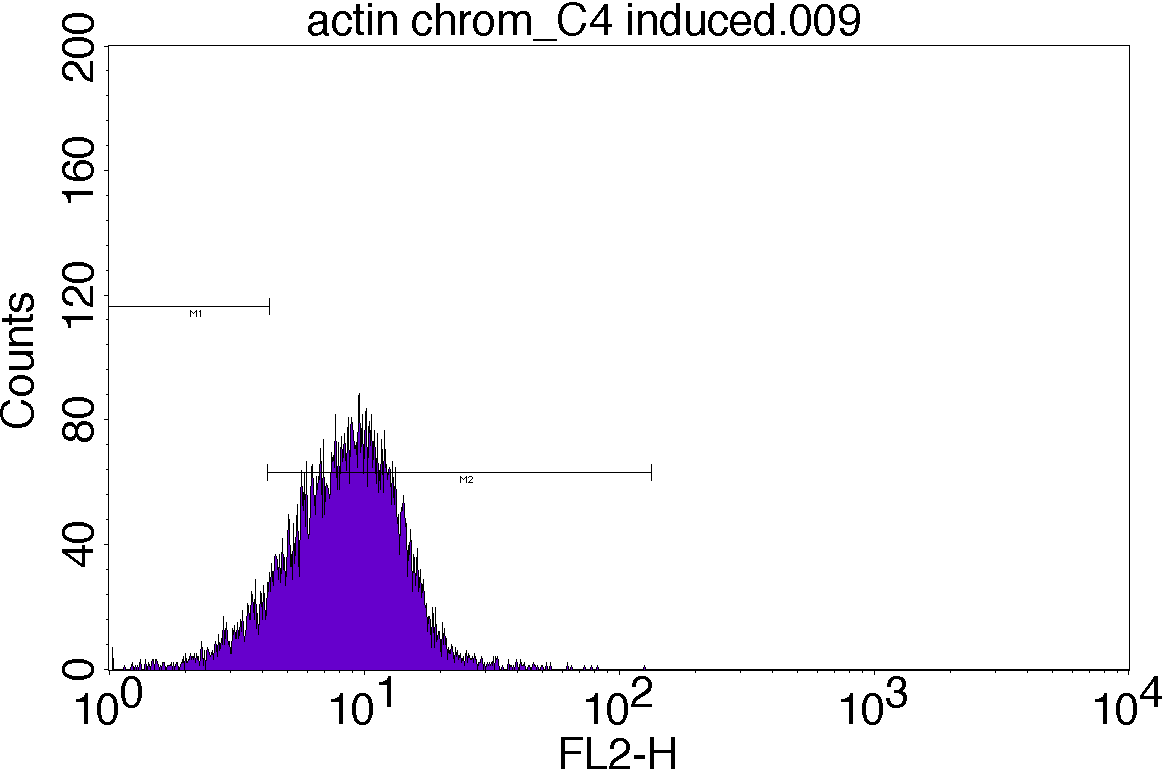

Supplement: Figure 6—figure supplement 1—source data 2. [file elife-96953-fig6-figsupp1-data2.zip › Figure6-Figure supplement1-Source data2/Panel C_Flow cytometry/actin chrom_C4 induced.cou.PNG]

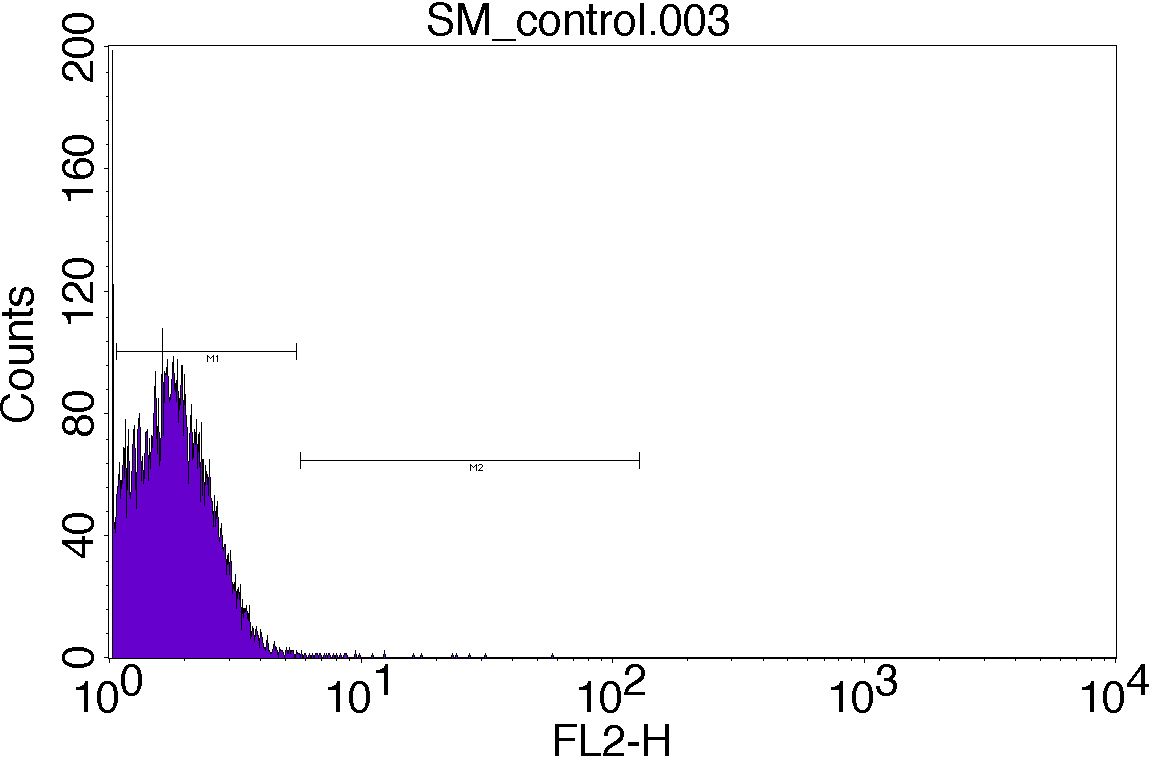

Supplement: Figure 6—figure supplement 1—source data 2. [file elife-96953-fig6-figsupp1-data2.zip › Figure6-Figure supplement1-Source data2/Panel C_Flow cytometry/SM_control.003_count.JPG]

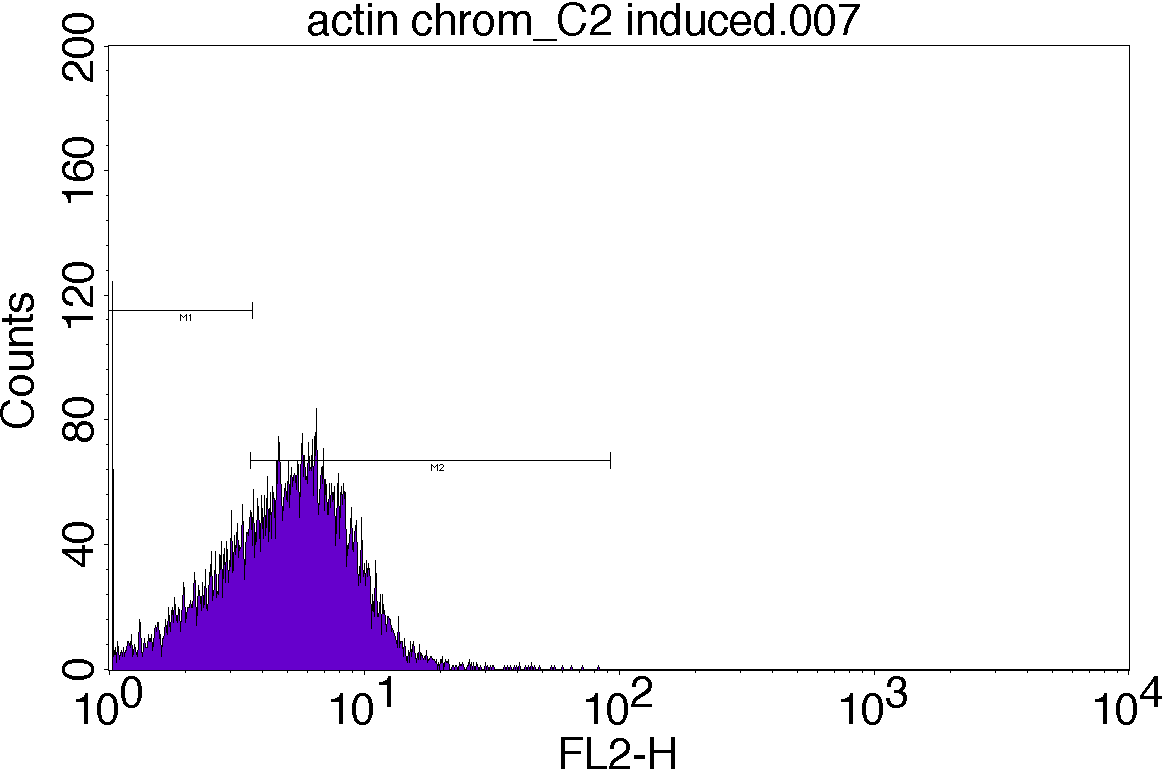

Supplement: Figure 6—figure supplement 1—source data 2. [file elife-96953-fig6-figsupp1-data2.zip › Figure6-Figure supplement1-Source data2/Panel C_Flow cytometry/actin chrom_C2 induced.cou.PNG]

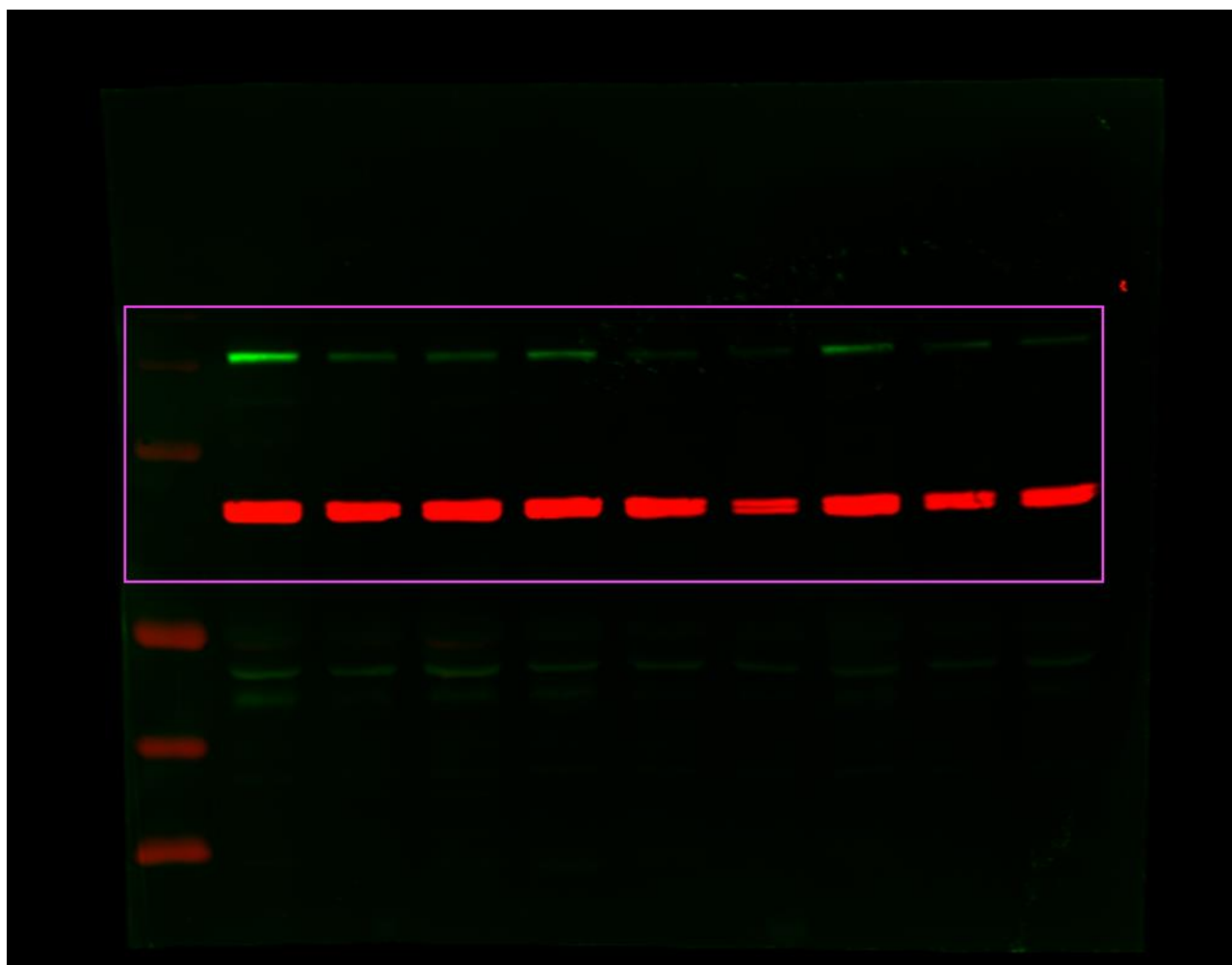

Panel B

Display area outlined in magenta

Supplement: Figure 7—figure supplement 1—source data 1. [file elife-96953-fig7-figsupp1-data1.zip › For zipping/Figure7-Figure supplement1-Source data1.pdf]

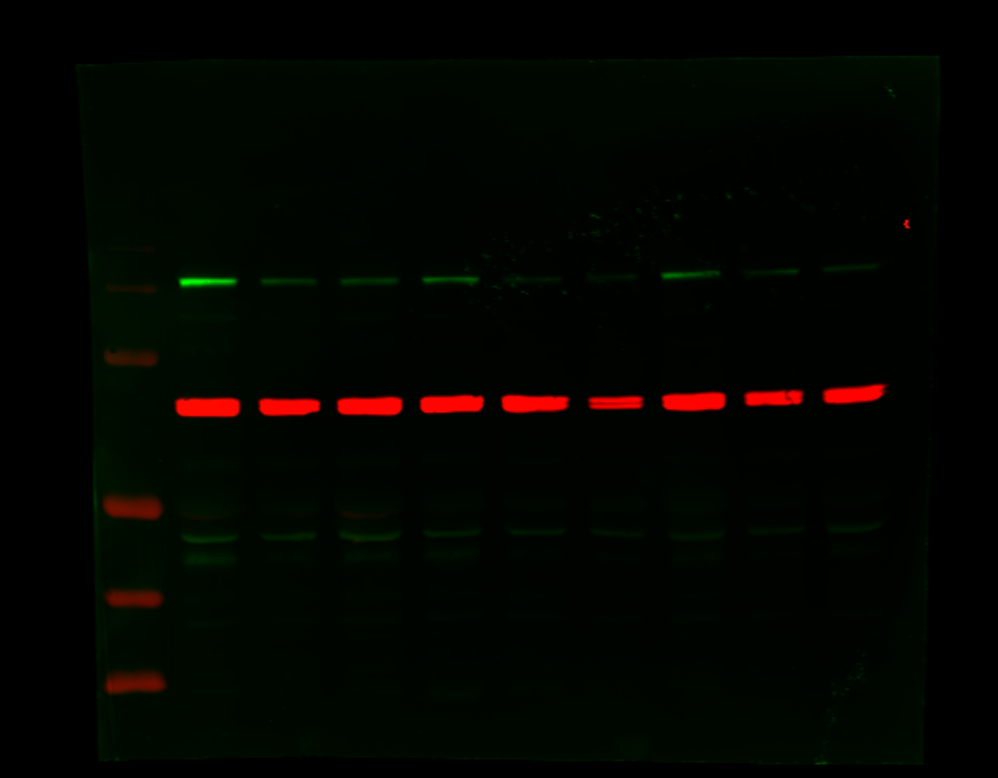

Supplement: Figure 7—figure supplement 1—source data 2. [file elife-96953-fig7-figsupp1-data2.zip › Figure7-Figure supplement1-Source data2/Panel B_Western_Blot_RNAi_t0_t24_t48_three_clones.png]
